# An image registration method for voxel-wise analysis of whole-body oncological PET-CT

**DOI:** 10.1038/s41598-022-23361-z

**Published:** 2022-11-05

**Authors:** Hanna Jönsson, Simon Ekström, Robin Strand, Mette A. Pedersen, Daniel Molin, Håkan Ahlström, Joel Kullberg

**Affiliations:** 1 Section of Radiology, Department of Surgical Sciences, Uppsala University, 751 85 Uppsala, Sweden; 2 Department of Information Technology, Uppsala University, 751 05 Uppsala, Sweden; 3 Department of Nuclear Medicine & PET-Centre, Aarhus University Hospital, 8200 Aarhus N, Denmark; 4 Department of Immunology, Genetics and Pathology, Uppsala University, 751 85 Uppsala, Sweden; 5 Antaros Medical AB, BioVenture Hub, 431 53 Mölndal, Sweden

**Keywords:** Image processing, Cancer imaging

## Abstract

Whole-body positron emission tomography-computed tomography (PET-CT) imaging in oncology provides comprehensive information of each patient’s disease status. However, image interpretation of volumetric data is a complex and time-consuming task. In this work, an image registration method targeted towards computer-aided voxel-wise analysis of whole-body PET-CT data was developed. The method used both CT images and tissue segmentation masks in parallel to spatially align images step-by-step. To evaluate its performance, a set of baseline PET-CT images of 131 classical Hodgkin lymphoma (cHL) patients and longitudinal image series of 135 head and neck cancer (HNC) patients were registered between and within subjects according to the proposed method. Results showed that major organs and anatomical structures generally were registered correctly. Whole-body inverse consistency vector and intensity magnitude errors were on average less than 5 mm and 45 Hounsfield units respectively in both registration tasks. Image registration was feasible in time and the nearly automatic pipeline enabled efficient image processing. Metabolic tumor volumes of the cHL patients and registration-derived therapy-related tissue volume change of the HNC patients mapped to template spaces confirmed proof-of-concept. In conclusion, the method established a robust point-correspondence and enabled quantitative visualization of group-wise image features on voxel level.

## Introduction

A large number of medical images are routinely collected in healthcare, but the information extracted from them in clinical routine is limited. For practical reasons, measurements are often reduced to selected regions of interest. This applies in particular to large images, such as whole-body positron emission tomography-computed tomography (PET-CT) and magnetic resonance imaging (MRI) scans. Global image features that are potentially relevant to patient outcome in diseases that involve the whole body, such as metabolic disorders and cancer, may be lost in the process. In clinical oncology, whole-body CT and PET are, in most cases, the first choices of imaging^1^. Images are most commonly acquired in combined PET-CT scanners with a field of view from mid-thigh to skull base with the patient positioned with arms up^2,3^. To take full advantage of oncological whole-body studies, methods that both enable the extraction of quantitative data from such datasets and streamline the analysis thereof, are needed.

Functional and anatomical differences in both diseased and non-diseased states have been studied extensively in neuroimaging by transforming images into a common coordinate system. This process, called image registration, forms the basis for voxel-wise statistics in statistical parametric mapping^4^ and deformation-based morphometry^5^. Other applications of image registration in medicine include, for example, fusion of images of different modalities (e.g. CT, MRI, and PET), attenuation correction in hybrid imaging systems, propagation of tissue labels between images for automatic segmentation of organs, and quantification of temporal changes through within-subject registration^6,7^. Registration algorithms are often designed specifically to the imaged body region and the imaging modality of their target application^8,9^. Most methods, basically, involve a search for the optimal alignment between two images in an iterative process and may be classified based on the choice of feature space, similarity metric, transformation type, and search strategy^10^. In recent years, the development of deep learning-based registration methods have accelerated. These methods, in general, offer faster runtimes and avoid problems specific to the optimization process of classical methods^11^. However, they often require large datasets to learn from to generate reliable results and newer methods without these requirements have only been evaluated on limited datasets^12,13^. Several domain-specific challenges need to be solved before deep learning-based registration methods can replace classical methods in a clinical settings^14^.

Image registration of whole-body images, both between different subjects and longitudinally within the same subject, is particularly challenging. Whole-body images may present with many different pathological findings or anatomical variants and are computationally expensive to process due to their size. Image registration algorithms targeted towards whole-body images must be able to alleviate these problems. Previously developed methods for whole-body image registration have been based on combinations of rigid and deformable steps^15–18^ or biomechanical models^19,20^. Prior studies on human whole-body CT image registration are, however, limited to within-subject registration of whole-body CT to PET^21,22^ or serial CT images from at most 30 subjects^16^. Between-subject registration of whole-body CT images to a common space where voxel-wise statistics can be performed remains, to our knowledge, largely unaddressed.

In this work, a registration method for whole-body (mid-thigh to skull base) PET-CT images was developed for spatial alignment and voxel-wise analysis of images from different subjects. In contrast to our related work on whole-body water-fat MRI image registration^17^, both low- and high-level features were used to establish the point-correspondence between the images. Low-level features were used in the form of the CT images with pixel intensities in Hounsfield units (HU). High-level features were used in the form of tissue segmentation masks generated from the CT images. This approach was hypothesized to be advantageous when the images to be registered are complex and may differ significantly from each other. Evaluation data included clinical PET-CT images of over 250 cancer patients from two disease cohorts. Finally, the registration method was used to provide voxel-wise visualizations of disease-related characteristics and thereby illustrate how image interpretation may be aided by computerized methods.

## Methods

### Image datasets

18^F^-fluorodeoxyglucose (FDG)-PET-CT images and clinical data from two separate datasets were used. The first dataset comprised baseline PET-CT images from a cohort of 157 patients with newly diagnosed classical Hodgkin lymphoma (cHL) treated at Uppsala University Hospital, Sweden. The images had been acquired on a whole-body PET-CT scanner according to standard protocol. Metabolic tumor volumes (MTVs) were segmented by a nuclear medicine physician using the ACCURATE software tool^23^. The MTVs were used to reduce inappropriate image distortion near lesions during image registration as described later. From the original dataset, 26 subjects were excluded due to inaccessible imaging data (n = 22) and arms-down positioning (n = 4). The final cHL dataset comprised baseline CT scans and MTVs of 131 subjects. Associated clinical data including age, height, and weight were available for a majority of them. Data collection and analysis were approved by the Swedish Ethical Review Authority and performed in accordance with the Declaration of Helsinki. Informed consent was obtained from all participants.

The second dataset comprised PET-CT image series from a collection of 215 patients with non-metastatic head and neck cancer (HNC) that is publically available at The Cancer Imaging Archive^24^. From the subset of 167 patients who had whole-body imaging before and after radiotherapy with inter-scan interval of less than 12 months, 32 subjects were excluded due to arms-down positioning (n = 30), separated abdominal and thoracic CT scans (n = 1), and inaccessible imaging data (n = 1). The final HNC dataset comprised baseline and follow-up CT scans, and associated clinical data^25^ of 135 subjects.

Patient data and key CT acquisition parameters of both datasets are presented in Table 1. Females comprised 49% (n = 64) of the cHL patients and 17% (n = 23) of the HNC patients. cHL patients had a mean age of 37 years (range, 8–79 years) while HNC patients had a mean age of 57 years (range, 35–91 years). Mean body weight and body mass index decreased between the pre- and post-therapy scans of the HNC patients. The CT scans of the cHL patients had lower in-plane axial resolution than the scans of the HNC patients.

**Table 1:** Patient and image characteristics.

	cHL	HNC
**Patient data**		
Total number, *n*	131	135
Females, *n*	64 (49%)	23 (17%)
Age (years)	37 ± 16 (8–79)*	57 ± 9 (35–91)
Height (cm)	173 ± 9 (146–197)**	174 ± 9 (146–197)
Pre-therapy weight (kg)	75 ± 16 (43–130)	86 ± 18 (45–145)
Post-therapy weight (kg)	N/A	77 ± 15 (41–111)***
Pre-therapy BMI (kg/m^2^)	25 ± 5 (15–42)**	29 ± 5 (18–44)
Post-therapy BMI (kg/m^2^)	N/A	25 ± 4 (16–40)***
**CT acquisition parameters**		
Kilovoltage peak (kVp)	123 ± 7 (100–140)	120 ± 0.0 (120–120)
Tube current (mA)	78 ± 28 (10–165)	264 ± 53 (58–300)
Slice thickness (mm)	3.65 ± 0.57 (0.62–5.0)	3.75 ± 0.0 (3.75–3.75)
Pixel spacing (mm × mm)	1.30 ± 0.15 (0.78–1.37)	0.98 ± 0.02 (0.98–1.37)
Image size, *x* × *y*	512 × 512	512 × 512
Axial slices, *z*	285 ± 145 (171–1283)	219 ± 16 (162–299)

Values are presented on the format mean ± standard deviation (min–max).

*BMI* body mass index, *cHL* classical Hodgkin lymphoma, *HNC* head and neck cancer.

*n = 122, **n = 130, ***n = 134 data available only of the post-therapy scans.

### Image registration

**Figure 1:**
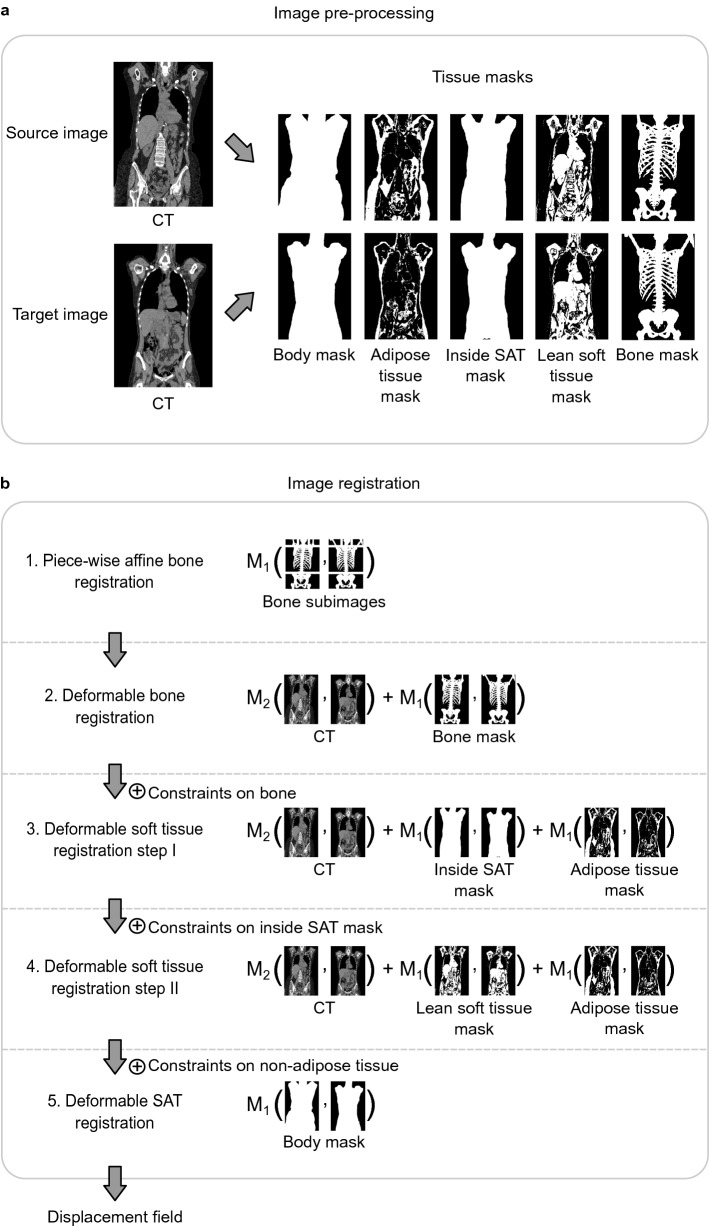
Image pre-processing (**a**) and registration steps (**b**). Image registration of one source image to one target image is illustrated. In (**a**), tissue masks are generated from both source and target image. In (**b**), these masks, are in addition to the original CT images, used to spatially align the images step-by-step. Image input, similarity metrics, and cost function used in each registration step is shown to the right. *M1* metric 1 (sum of squared differences), *M2* metric 2 (Pearson’s correlation coefficient), *SAT* subcutaneous adipose tissue.

Figure 1 illustrates the registration process for one pair of images. One image is referred to as the source image and the other is referred to as the target image. The final output of the registration pipeline is the displacement field that spatially aligns the source image with the target image.

#### Image pre-processing

Five masks were generated from each CT image for use as input during registration. These masks are illustrated to the right in Fig. 1a for one source and one target image.

Body masks were first generated to remove the CT table and other non-body objects from all CT images before any further processing. These masks were generated through a combination of CT thresholding, morphological operations, and the output of a U-Net^26^ trained on a subset of high-quality body masks to remove CT table portions connected to the body.

Three tissue masks were defined by CT intensity ranges in HU, representing bone ($>$+ 200 HU)^27^, lean soft tissue (− 29 to + 150 HU)^28^, and adipose tissue (− 190 to − 30 HU)^29,30^. From these tissue masks, a mask comprising the body region enclosed by subcutaneous adipose tissue (SAT) was generated with an in-house developed algorithm. The resulting mask is referred to as an inside SAT mask.

Before registering any image to another, both images were resampled to the largest voxel spacing of the two, if there was a resolution difference greater than a defined tolerance level ($\epsilon$ = 0.1 mm) applied to the x, y and z-axes separately.

#### Registration method

An image registration pipeline was built to spatially align images step-by-step. Constraints were successively introduced between the steps to lock the displacement of already aligned tissues. One affine registration step and four deformable registration steps were implemented. The steps are illustrated in Fig. 1b.

In the first registration step, a piece-wise affine registration of the skeleton was performed using bone subimages as input and sum of squared differences as similarity metric. The affine transform parameters were restricted to include no shear factors. First, the bone mask cropped to torso range was registered to yield initial transform parameters. The torso bone mask was subsequently cropped into five subregions (right arm, head, left arm, chest and pelvis) that were registered separately to generate subregion-specific updated transform parameters. A piece-wise affine transform of the whole skeleton was constructed from the subregion parameters using Gaussian smoothing. This transform was used to initialize the first deformable registration step described below.

Next, deformable registration was performed in four steps. A weighted cost function that combined multiple image channels and multiple similarity metrics was used in all except the last deformable registration step. The image channels and similarity metrics that defined the cost function in each step are illustrated to the right in Fig. 1b. Pearson’s correlation coefficient was used a similarity metric between CT image channels and sum of squared differences was used as similarity metric between tissue masks. In the first deformable registration step, the CT image and bone mask were used as image input to complete the alignment of bone. Bone voxels were then constrained before registration of soft tissues in two steps. The first soft tissue registration step used the CT image, inside SAT mask, and adipose tissue mask as input. Constraints were then put on voxels along the outer boundary of the inside SAT mask, before a refined second registration of soft tissue was performed using the CT image, lean soft tissue mask, and adipose tissue mask as input. Finally, constraints were put on non-adipose tissue, excluding the skin, before the body mask was used as input in the last registration step that completed the alignment of SAT.

The registration pipeline was implemented in Python. Affine registration was performed with the SimpleElastix library^31^ and deformable registration was performed with a GPU-accelerated graph-cut based method^32,33^. All steps used a multi-resolution strategy with Gaussian image pyramids, where level $i$ of the pyramid represents an image downsampled by a factor of ${2}^{i}$ in each coordinate direction. Registration was in each step initialized at level 4 and terminated after level 2. Tissue-specific regularization weights were applied in deformable registration with a higher regularization weight applied to tissues with low elasticity (e.g. bone) than to tissues with high elasticity (e.g. adipose tissue). Registration was performed on a desktop computer (64-bit Windows, Intel(R) Xeon(R) W-2102 quad-core CPU 2.90 GHz, 32 GB RAM) with an Nvidia GeForce GTX 1080 Ti graphics card.

#### Between-subject registration to template space

Between-subject registration was performed of the cHL images and pre-therapy HNC images. Female and male subsets of each dataset were registered separately to a common coordinate space (template space) defined by one image in each subset. Template spaces (subjects) were chosen to meet two criteria; a neutral image positioning and fat body mass close to the median of its subset. Image fat percentage was calculated inside the body mask and used as a surrogate measure of fat body mass for the cHL dataset while available clinical data of pre-therapy CT-derived fat body mass was used for the HNC dataset.

Cost function masking was applied to the cHL images to limit registration bias near tumor regions^34^. Masks that defined voxels that contributed less to the matching cost were constructed for all images by applying a Gaussian filter with a standard deviation of three millimeters to the MTV segmentations.

#### Within-subject registration

Within-subject registration of the HNC images was performed with the same method as between-subject registration. Each subject’s post-therapy scan was registered to its pre-therapy scan.

### Evaluation

The performance of the registration method was evaluated in terms of the resulting transformed images, transform properties, and ability to visualize disease state characteristics of the patient subsets on voxel level. Performance metrics were based on a standardized set suggested for evaluation of non-rigid image registration algorithms^35^. Transformed images were during the development phase reviewed individually to visually assess their quality.

#### Template space statistics

Voxel-wise CT statistics (median and interquartile range) were computed of all registered images, in each template space separately. Voxel-wise mean intensity absolute errors between the registered images and their corresponding template space CT scan were also computed.

#### Transform properties

Both between- and within-subject registration were performed in reversed direction to evaluate the consistency of the mapping produced by the registration algorithm. Ideally, there should be a consistent correspondence between the points in any images, such that the correspondence defined by the forward transform ${T}_{ij}$ of image $i$ to image $j$ is the same as that of the inverse transform ${T}_{ji}$. Inverse consistency errors were defined in terms of average vector and intensity magnitude errors (VME, IME) over segmented body regions $\Omega$, as follows,

$$VM{E}_{ij}=\frac{1}{\left|\Omega \right|}\sum_{x\in \Omega }||x-{T}_{ji}\circ {T}_{ij}\left(x\right)||,$$

$$IM{E}_{ij}=\frac{1}{\left|\Omega \right|}\sum_{x\in \Omega }||{I}_{i}\left(x\right)-{I}_{i}\left({T}_{ji}\circ {T}_{ij}\left(x\right)\right)||,$$

where ${I}_{i}\left(x\right)$ is the intensity value of image $i$ at voxel location $x$. The inverse consistency errors of composite transforms ${T}_{ij}\circ {T}_{ji}$ and ${T}_{ji}\circ {T}_{ij}$ were averaged into one value per pair of images $i$ and $j$ that were registered to each other. Similarly, the spatial overlap between the original tissue masks and those resulting after applying the composite transforms was evaluated with the Dice similarity coefficient. Dice coefficients were computed on adipose tissue and lean soft tissue. The Jacobian determinants of all forward and inverse transforms were examined for regions with negative values (folding). Total registration time excluding the pre-processing steps was measured per image.

#### Voxel-wise applications

**Figure 2:**
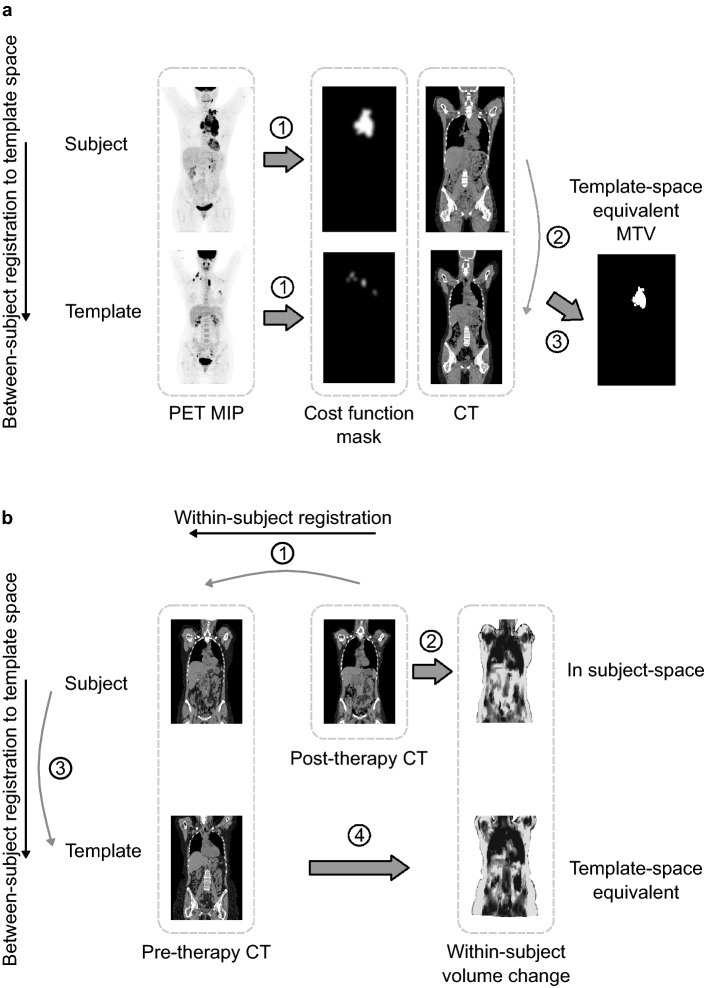
Application of the image registration method to the images of (**a**) classical Hodgkin lymphoma (cHL) and (**b**) head and neck cancer (HNC) patients. Each subfigure shows the output of a single subject and was repeated for all subjects in each dataset respectively. In (**a**), each cHL subject’s CT image is first registered to the template subject’s CT with cost function masking using the MTV segmentations to define voxels that contributed less to the matching cost. Next, each subject’s MTV is transferred to template space using the transform from between-subject registration to generate a template-space equivalent MTV. In (**b**), each HNC subject’s post-therapy CT scan is first registered to its pre-therapy scan. The Jacobian determinant (local volume change map) of the resulting within-subject transform is then generated. Next, the subject’s pre-therapy scan is registered to the template subject’s pre-therapy scan and the resulting between-subject transform is used to generate a template-space equivalent local volume change map. *MIP* maximum intensity projection, *MTV* metabolic tumor volume.

Between- and within-subject registration were further evaluated in terms of ability to summarize and visualize quantifiable image features on voxel-level. Figure 2 illustrates how the registration method was applied to generate voxel-wise feature maps of the two patient cohorts.

Figure 2a shows the output generated from each subject of the cHL subset. A template-space equivalent MTV was generated for each subject of the cHL subset using the results from between-subject registration according to above. After transferring all original MTV segmentations of the cHL subset to template-space equivalent MTVs, the number of tumor occurrences were summarized voxel-wise in the female and male template spaces and normalized with the number of subjects in the subsets respectively. This results in female and male tumor distribution maps in which each voxel value represents the percentage of subjects with tumor occurrence at that location in the body.

Figure 2b shows the output generated from each subject of the HNC subset. A local volume change map was derived for each subject of the HNC subset using the results from within-subject transform according to above. The local volume change is found from the Jacobian determinant of the transform. Whole-body volume change was derived from this map by summarizing the volume change of all body voxels. The strength of the association between this variable and the actual change in body weight between the scans was evaluated with Pearson’s correlation coefficient. Log transformation of both variables was applied in the analysis^36^. All local volume change maps of the HNC subset were also transferred to template-space equivalent maps^37^ using the results from between-subject registration according to above. These maps were then averaged voxel-wise in the template spaces to create the local average volume factor of all registered female and male subjects respectively. To correct for local folding, all voxels with a value of less than $q=0.05$ were assigned the value $q$ before averaging.

## Results

**Figure 3:**
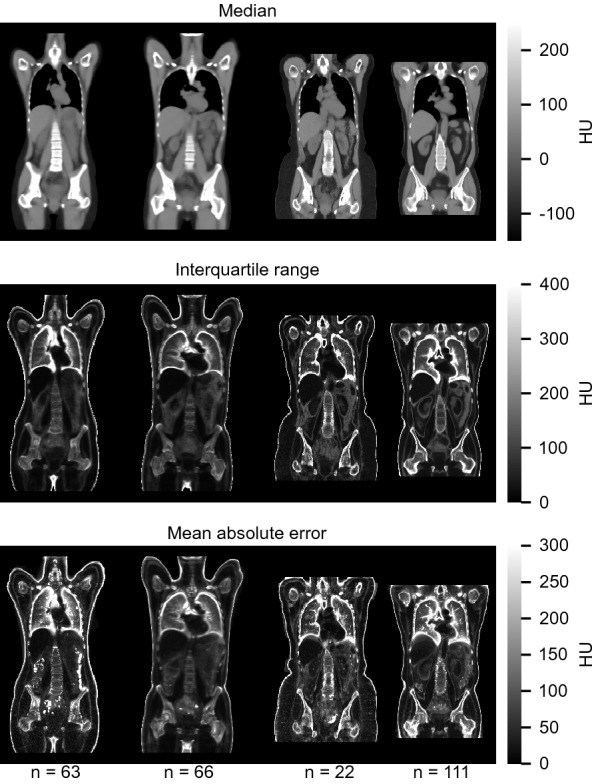
Mid-coronal planes of voxel-wise CT statistics of images registered to template spaces. Each panel shows the results from registered female cHL images, male cHL images, female HNC images and male HNC images from left to right. The number of subjects in each subset is shown below the bottom figure. A soft tissue window is used to display the top panel. *HU* Hounsfield units.

Figure 3 shows mid-coronal planes of the voxel-wise CT statistics computed of all images after registration to the four template spaces. Additional axial, coronal, and sagittal planes of the statistical images are provided as Supplementary Videos S1–S3, S4–S6 and S7–S9 respectively. In the median intensity images (Fig. 3, top panel and Supplementary Videos S1, S4 and S7), major organs and anatomical structures were identified. Intensity variation was highest at borders between different organs (Fig. 3, middle panel and Supplementary Videos S2, S5 and S8), especially near the left and right hemidiaphragms of the HNC subsets. These regions were also identified to have among the highest mean absolute errors across the body (Fig. 3, bottom panel and Supplementary Videos S3, S6 and S9) together with image regions of inconsistent use of contrast agents and where metallic implants were present. Masked regions during registration of the cHL images further caused an intensity offset, visible, for example, along the right side of the neck in the statistical images of the female cHL subset. Misalignment of subcutaneous adipose tissue was mostly noted in the abdominal region and breast tissue, and could be attributed to a few obese subjects.

**Figure 4:**
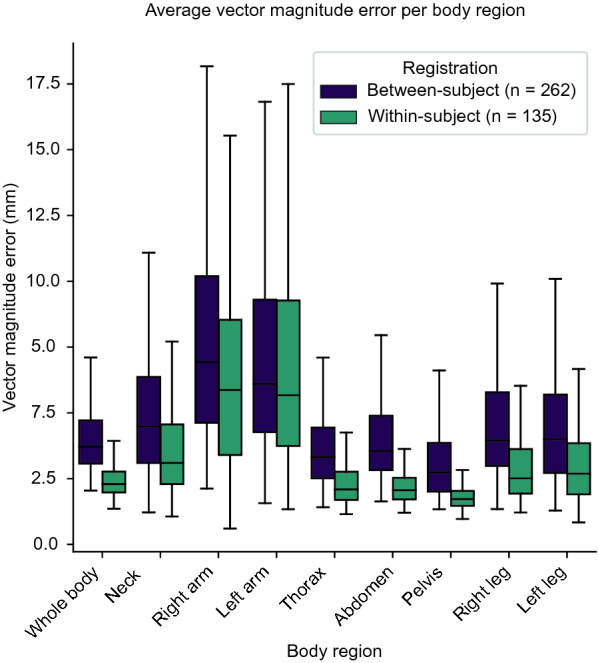
Box plots of average vector magnitude error computed per body region and registration task.

Vector magnitude errors per body region and registration task are presented in Fig. 4. The median error was higher in between- than in within-subject registration in all studied body regions. Thoracic, abdominal and pelvic regions were associated with lower errors than more peripheral image regions.

Table 2 shows the mean, median, and standard deviation of the inverse consistency errors computed inside whole bodies per registration task. Percentages of within-body voxels with folding and total registration times are also presented in Table 2. The mean vector magnitude error was less than 5 and 3 mm in between- and within-subject registration respectively. The mean intensity magnitude error was less than 45 HU, Dice coefficients of adipose tissue and lean soft tissue were above 0.85, and the percentage of folding was less than 0.5% in both tasks. The mean registration time was 15 and 10 min in between- and within-subject registration respectively. Within-subject registration achieved the best results with respect to all performance metrics.

**Table 2:** Whole-body registration performance metrics.

	Between-subject registration (*n* = 262)	Within-subject registration (*n* = 135)
**Inverse consistency errors**		
VME (mm)	4.3 (3.7) ± 2.2	2.6 (2.3) ± 1.0
IME (HU)	44 (44) ± 15	34 (32) ± 7
Dice coefficient, adipose tissue	0.87 (0.88) ± 0.04	0.89 (0.91) ± 0.04
Dice coefficient, lean soft tissue	0.89 (0.89) ± 0.05	0.91 (0.92) ± 0.02
**Folding (%)**	0.1 (0.0) ± 0.2	0.0 (0.0) ± 0.1
**Total registration time (min)**	15.6 (13.4) ± 6.3	10.4 (9.9) ± 2.8
Affine bone registration	0.7 (0.7) ± 0.3	0.5 (0.4) ± 0.1
Deformable bone registration	5.3 (4.5) ± 2.8	3.4 (3.0) ± 1.5
Deformable soft tissue registration step I	4.1 (3.5) ± 1.9	2.6 (2.5) ± 0.9
Deformable soft tissue registration step II	3.6 (3.0) ± 1.7	2.5 (2.3) ± 1.0
Deformable SAT registration	2.0 (1.7) ± 1.1	1.4 (1.3) ± 0.6

Values are presented on the format mean (median) ± standard deviation.

*VME* vector magnitude error, *IME* intensity magnitude error, *HU* Hounsfield units, *SAT* subcutaneous adipose tissue.

**Figure 5:**
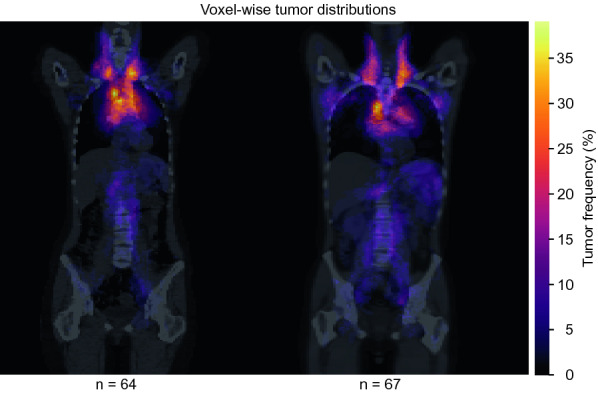
Metabolic tumor volume distributions derived from between-subject registration of classical Hodgkin lymphoma patients. Distributions are presented as maximum intensity projections overlaid in color on mid-coronal planes of the chosen female (left) and male (right) template CTs. The number of subjects in each subset is shown below the figure.

Figure 5 shows voxel-wise spatial MTV distributions of the cHL patients in female and male template spaces. High involvement of the mediastinum and neck lymph nodes is seen.

**Figure 6:**
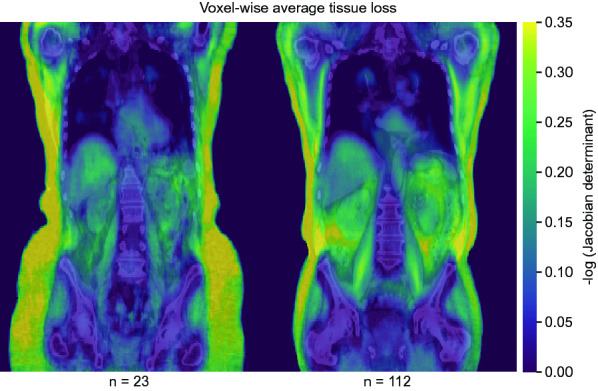
Average tissue loss between pre- and post-therapy CT scans derived from between- and within-subject registration of head and neck cancer patients. Mid-coronal planes are shown overlaid in color on the chosen female (left) and male (right) template CTs. The number of subjects in each subset is shown below the figure.

Within-subject registration derived whole-body volume change correlated with actual body weight change for the female (R = 0.87, p < 0.001) and male (R = 0.86, p < 0.001) subsets of the HNC patients. Figure 6 shows the voxel-wise average tissue loss (reversed log Jacobians $>$ 0) between the pre- and post-therapy scans of the same cohort in female and male template spaces. Loss of predominantly adipose tissue and muscle mass is visible.

## Discussion

In this work, an image registration method for whole-body PET-CT images was evaluated. The method performed, in general, satisfactorily when applied to over 250 clinical images. In more detail, average vector magnitude errors were less than twice the average slice thickness and less than approximately five pixels in the axial plane for both between- and within-subject registration. Image registration of each of the included datasets was performed within hours to days, which was considered feasible in time. In terms of applicability, a proof of concept was demonstrated by two applications of the registration method. First, presented spatial tumor distributions of the cHL patients were consistent with the most frequent presentation of cHL^38^. Second, the registration method was able to capture adipose tissue and muscle mass loss of the HNC patients from pre- and post-therapy images. Together, the voxel-wise applications illustrated that the method was able to quantify and visualize group-wise image features.

Several challenges related to between-subject registration of brain images exists and were also encountered in this study of whole-body image registration. These challenges often include between-subject variability, missing correspondence, influences of intensity inhomogeneities, and field of view differences^39^. Each of these topics will now be discussed in the context of our study.

Between-subject variability can lead to difficulties in finding point-correspondence between images of different subjects. Here, such difficulties were evidenced by higher inverse consistency errors in between- than in within-subject registration. However, our study also provided some insight into how such errors may be reduced. A common feature between the presented method and previously described whole-body registration methods applied to images of mice and humans^15–18^, is the use of several steps, starting with a registration of the skeleton. In this work, however, soft tissue registration was divided into two sub-steps, where the first sub-step was especially seen to improve between-subject registration of subjects that differed profoundly from the templates in terms of body composition, mostly fat mass. When source and target images were not already very similar from the beginning, the benefit of adding the last registration step that finalized the alignment of subcutaneous adipose tissue was also found greatest. However, when more similar images were registered to each other, preceding registration steps were often able to complete the alignment. This suggests that multiple registration steps are important to guide the registration when images are increasingly different, to avoid termination of registration in local minima.

Tumor regions of the cHL patients and also the presence of various medical devices contributed to missing correspondence in the images. Voxels with missing correspondence had increased intensity variation and absolute error in template spaces. However, the registration process was considered robust to the presence of such objects as these were generally found registered to corresponding anatomical locations. Furthermore, intensity inhomogeneities present in the images due to, for example, varying use of contrast agents, were not found to deteriorate results. The influences of missing correspondence and intensity inhomogeneities on registration performance is supposed to have been minimized by having a correlation part in the weighted cost function that is robust to linear intensity changes.

Field of view differences and non-standardized arm positioning likely explains that vector magnitude errors were larger in the head and arm regions than in other body regions. The field of view of the images in this study was smaller than that of the images used in our previous evaluation of a registration method for whole-body MRI^17^, but, more importantly, varied notably between subjects. Furthermore, the images of the present study were of higher resolution than in our previous study. This may contribute to any differences in performance metrics seen between the two studies, but also makes it difficult to directly compare the results. Nevertheless, the present study achieved similar performance to our previous and our applied studies on whole-body MRI have demonstrated this level of registration accuracy to be enough to derive voxel-wise cohort associations and perform multi-parametric analysis^40,41^.

A trade-off between minimizing the intensity error between images and generating smooth deformation fields was noted during preliminary studies. Failure to align very obese subjects completely to template spaces was found to arise at least in part due to regularization constraints. However, reducing the regularization level increased the presence of unrealistic tissue deformations. It is also possible that registration errors located to the hemidiaphragms are the results of larger regularization constraints on lean soft tissue than on lung tissue (air). Since voxel-wise constraints were source image-specific, the level of regularization may in some cases better be based on both source and target images. This study did not quantitatively compare voxel-wise constraints to a global level of regularization, but, in other work, adaptive regularization based on image similarity between source and target images was superior to using a fixed level^42^. In our study, low levels of folding suggests no general problems with underconstrained transformations.

This study has several strengths. First, this study was, to our knowledge, the first to apply and evaluate between-subject registration of whole-body PET-CT images of over one hundred human subjects. Second, the study included multi-center image data from two patient populations with different characteristics in terms of, for example, age and body composition. Third, no exclusion criteria other than arm positioning was used. Accordingly, the registration method was evaluated on a material with commonly occurring patient presentations represented. Results should therefore generalize well to a clinical setting. Fourth, the registration method was designed to be almost fully automatic with little manual input required, as this is important for a method to scale well with larger datasets.

This study has limitations. First, the absence of directly comparable studies makes it difficult to relate our results to previous work. No established reference method for whole-body image registration exists. A basic registration based on, for example, affine registration only, would clearly not be comparable with more sophisticated methods such as the one presented in this study. Second, the presented voxel-wise applications are difficult to evaluate quantitatively due to a lack of available ground truth at voxel level. A proof of concept was instead illustrated by anatomically plausible results that also agreed with clinical expectations. Third, the use of thresholding to generate tissue segmentation masks is likely to result in inconsistent masks when applied to both images with and without contrast enhancement. The multi-channel weighted cost function is supposed to have reduced the impact of such errors, but more advanced methods than thresholding may be used to improve the masks. Fourth, our study was limited to whole-body images according to the most commonly defined field of view and thus did not include true whole-body images (top of head to bottom of feet). However, the described method is expected to be applicable to true whole-body images after minor adjustments to the registration steps. Fifth, the smooth deformation fields imposed by the regularization constraints during registration cannot account for small structural variations. While this may not be limiting in studies of global imaging patterns, it does limit the scale at which details can be discovered and will be an important aspect to address in applied studies.

In conclusion, we have presented a step-by-step image registration method that combined low-level features from CT images with high-level features from tissue segmentation masks. We demonstrated that the method was feasible to apply on a large clinical dataset and was found suitable for both between- and within-subject registration. The method may serve as a starting point for more automated cross-sectional and longitudinal voxel-wise analysis of whole-body PET-CT images. The benefit of this approach is that the whole field of view may be explored to find which image regions are most informative. Possible applications include studies of tumor distribution or body composition and its association to patient outcome. The method may also be used for therapy response evaluations through within-subject registration of baseline and follow-up images.

## Supplementary Information

Supplementary Legends.

Supplementary Video S1.

Supplementary Video S2.

Supplementary Video S3.

Supplementary Video S4.

Supplementary Video S5.

Supplementary Video S6.

Supplementary Video S7.

Supplementary Video S8.

Supplementary Video S9.

## Data Availability

The classical Hodgkin lymphoma dataset analyzed during the current study is available from the corresponding author on reasonable request. The head and neck cancer dataset analyzed during the current study is available in The Cancer Imaging Archive repository, HNSCC [Dataset]: 10.7937/k9/tcia.2020.a8sh-7363.

## References

[1] Ehman EC (2017). PET/MRI: Where might it replace PET/CT?. J. Magn. Reson. Imaging.

[2] Delbeke D, Schöder H, Martin WH, Wahl RL (2009). Hybrid imaging (SPECT/CT and PET/CT): Improving therapeutic decisions. Semin. Nucl. Med..

[3] Von Schulthess GK, Steinert HC, Hany TF (2006). Integrated PET/CT: Current applications and future directions. Radiology.

[4] Friston KJ (1994). Statistical parametric maps in functional imaging: A general linear approach. Hum. Brain Mapp..

[5] Ashburner J (1998). Identifying global anatomical differences: Deformation-based morphometry. Hum. Brain Mapp..

[6] Sotiras A, Davatzikos C, Paragios N (2013). Deformable medical image registration: A survey. IEEE Trans. Med. Imaging.

[7] Iglesias JE, Sabuncu MR (2015). Multi-atlas segmentation of biomedical images: A survey. Med. Image Anal..

[8] Maintz JBA, Viergever MA (1998). A survey of medical image registration. Med. Image Anal..

[9] Hajnal JV, Hill DLG (2001). Medical Image Registration.

[10] Oliveira FPM, Tavares JMRS (2014). Medical image registration: A review. Comput. Methods Biomech. Biomed. Eng..

[11] Boveiri HR, Khayami R, Javidan R, Mehdizadeh A (2020). Medical image registration using deep neural networks: A comprehensive review. Comput. Electr. Eng..

[12] Haskins G, Kruger U, Yan P (2020). Deep learning in medical image registration: A survey. Mach. Vis. Appl..

[13] Fu Y (2020). Deep learning in medical image registration: a review. Phys. Med. Biol..

[14] Chen X, Diaz-Pinto A, Ravikumar N, Frangi AF (2021). Deep learning in medical image registration. Prog. Biomed. Eng..

[15] Li X, Yankeelov TE, Peterson TE, Gore JC, Dawant BM (2008). Automatic nonrigid registration of whole body CT mice images. Med. Phys..

[16] Akbarzadeh A (2013). Evaluation of whole-body MR to CT deformable image registration. J. Appl. Clin. Med. Phys..

[17] Strand R (2017). A concept for holistic whole body MRI data analysis Imiomics. PLoS ONE.

[18] Baiker M, Staring M, Löwik CWGM, Reiber JHC, Lelieveldt BPF, Fichtinger Gabor, Martel Anne, Peters Terry (2011). Automated registration of whole-body follow-up MicroCT data of mice. Medical Image Computing and Computer-Assisted Intervention (MICCAI).

[19] Li M (2015). Patient-specific biomechanical model as whole-body CT image registration tool. Med. Image Anal..

[20] Li M, Miller K, Joldes GR, Kikinis R, Wittek A (2016). Biomechanical model for computing deformations for whole-body image registration: A meshless approach. Int. J. Numer. Method Biomed. Eng..

[21] Slomka PJ (2003). Automated 3-dimensional registration of stand-alone 18 F-FDG whole-body PET with CT. J. Nucl. Med..

[22] Shekhar R (2005). Automated 3-dimensional elastic registration of whole-body PET and CT from separate or combined scanners. J. Nucl. Med..

[23] Flechsig P (2018). Role of CT density in PET/CT-based assessment of lymphoma. Mol. Imaging Biol..

[24] Clark K (2013). The cancer imaging archive (TCIA): Maintaining and operating a public information repository. J. Digit. Imaging.

[25] Grossberg AJ (2018). Imaging and clinical data archive for head and neck squamous cell carcinoma patients treated with radiotherapy. Sci. Data.

[26] Ronneberger O, Fischer P, Brox T, Navab Nassir, Hornegger Joachim, Wells William M, Frangi Alejandro F (2015). U-net: Convolutional networks for biomedical image segmentation. Medical Image Computing and Computer-Assisted Intervention (MICCAI).

[27] Broder J, Broder J (2011). Imaging of nontraumatic abdominal conditions. Diagnostic Imaging for the Emergency Physician.

[28] Mitsiopoulos N (1998). Cadaver validation of skeletal muscle measurement by magnetic resonance imaging and computerized tomography. J. Appl. Physiol..

[29] Heymsfield SB, McManus CB (1985). Tissue components of weight loss in cancer patients. A new method of study and preliminary observations. Cancer.

[30] Kvist H, Sjostrom L, Tylen U (1986). Adipose tissue volume determinations in women by computed tomography: Technical considerations. Int. J. Obes..

[31] Marstal, K., Berendsen, F., Staring, M. & Klein, S. SimpleElastix: A user-friendly, multi-lingual library for medical image registration. In *IEEE Computer Society Conference on Computer Vision and Pattern Recognition Workshops* 574–582 (IEEE, 2016), 10.1109/CVPRW.2016.78.

[32] Ekström S, Malmberg F, Ahlström H, Kullberg J, Strand R (2020). Fast graph-cut based optimization for practical dense deformable registration of volume images. Comput. Med. Imaging Graph..

[33] Ekström S (2021). Faster dense deformable image registration by utilizing both CPU and GPU. J. Med. Imaging.

[34] Brett M, Leff AP, Rorden C, Ashburner J (2001). Spatial normalization of brain images with focal lesions using cost function masking. Neuroimage.

[35] Christensen GE, Christensen GE (2006). Introduction to the Non-rigid Image Registration Evaluation Project (NIREP). Biomedical Image Registration.

[36] Leow AD (2007). Statistical properties of Jacobian maps and the realization of unbiased large-deformation nonlinear image registration. IEEE Trans. Med. Imaging.

[37] Ridgway GR, Leung KK, Ashburner J, Ridgway GR, Leung KK, Ashburner J (2015). Computing brain change over time. Brain Mapping: An Encyclopedic Reference.

[38] Sharma A (2004). Patterns of lymphadenopathy in thoracic malignancies. Radiographics.

[39] Ou Y, Akbari H, Bilello M, Da X, Davatzikos C (2014). Comparative evaluation of registration algorithms in different brain databases with varying difficulty: Results and insights. IEEE Trans. Med. Imaging.

[40] Lind L, Strand R, Michaëlsson K, Ahlström H, Kullberg J (2020). Voxel-wise study of cohort associations in whole-body MRI: Application in metabolic syndrome and its components. Radiology.

[41] Sjöholm T (2019). A whole-body FDG PET/MR atlas for multiparametric voxel-based analysis. Sci. Rep..

[42] Simpson IJA, Schnabel JA, Groves AR, Andersson JLR, Woolrich MW (2012). Probabilistic inference of regularisation in non-rigid registration. Neuroimage.

