# Supplementary material for: An image registration method for voxel-wise analysis of whole-body oncological PET-CT
Source: Sci Rep. 2022 Nov 5;12:18768. doi: 10.1038/s41598-022-23361-z (PMC9637131; doi:10.1038/s41598-022-23361-z)
Supplement: Supplementary file 1 — Supplementary Legends. [file 41598_2022_23361_MOESM1_ESM.pdf]

An image registration method for voxel-wise analysis of whole-body oncological PET-CT

Hanna Jönsson, Simon Ekström, Robin Strand, Mette A. Pedersen, Daniel Molin, Håkan Ahlström, Joel Kullberg

Supplementary Information

Supplementary Video S1. Axial planes of the voxel-wise median of CT images registered to template spaces. The results of registered female cHL images, male cHL images, female HNC images, and male HNC images are shown from left to right with the number of subjects in each subset below. A soft tissue window is used for display. HU = Hounsfield units

Supplementary Video S2. Axial planes of the voxel-wise interquartile range of CT images registered to template spaces. The results of registered female cHL images, male cHL images, female HNC images, and male HNC images are shown from left to right with the number of subjects in each subset below. HU = Hounsfield units

Supplementary Video S3. Axial planes of the voxel-wise mean absolute error of CT images registered to template spaces. The results of registered female cHL images, male cHL images, female HNC images, and male HNC images are shown from left to right with the number of subjects in each subset below. HU = Hounsfield units

Supplementary Video S4. Coronal planes of the voxel-wise median of CT images registered to template spaces. The results of registered female cHL images, male cHL images, female HNC images, and male HNC images are shown from left to right with the number of subjects in each subset below. A soft tissue window is used for display. HU = Hounsfield units

Supplementary Video S5. Coronal planes of the voxel-wise interquartile range of CT images registered to template spaces. The results of registered female cHL images, male cHL images, female HNC images, and male HNC images are shown from left to right with the number of subjects in each subset below. HU = Hounsfield units

Supplementary Video S6. Coronal planes of the voxel-wise mean absolute error of CT images registered to template spaces. The results of registered female cHL images, male cHL images, female HNC images, and male HNC images are shown from left to right with the number of subjects in each subset below. HU = Hounsfield units

Supplementary Video S7. Sagittal planes of the voxel-wise median of CT images registered to template spaces. The results of registered female cHL images, male cHL images, female HNC images, and male HNC images are shown from left to right with the number of subjects in each subset below. A soft tissue window is used for display. HU = Hounsfield units

Supplementary Video S8. Sagittal planes of the voxel-wise interquartile range of CT images registered to template spaces. The results of registered female cHL images, male cHL images, female HNC images, and male HNC images are shown from left to right with the number of subjects in each subset below. HU = Hounsfield units

Supplementary Video S9. Sagittal planes of the voxel-wise mean absolute error of CT images registered to template spaces. The results of registered female cHL images, male cHL images, female HNC images, and male HNC images are shown from left to right with the number of subjects in each subset below. HU = Hounsfield units
